# Medical Schools in the United States

**Published:** 1890-11-15

**Authors:** 


### Medical Schools in the United States.

Our cousins across the Atlantic have, in relation to the regulation of medical practice, and the education of physicians, some ways and methods which seem a little strange to us, although they would not have been considered very remarkable by English physicians of the commencement of this century.

It must be admitted, however, that in most respects they seem to be getting there just the same.

There are now about 100 regular medical schools in the United States, besides those devoted to particular pathies, or isms. Of these about one half require attendance on three or more courses of lectures as a condition of graduation, the remainder requiring a two years' course only.

Almost all of them profess to exact certain educational qualifications of their matriculants, but only about a dozen require more than a fair common school education, and the great majority demand little more than that the candidate shall be able to read and write.

All of these schools except five confer the degree of M.D., by State authority. The Federal Government exercises no control over the practice of medicine or the requirements of Medical Education in the country with the possible exception of the District of Columbia where its authority is supreme. The regulation of medical education and practice pertains to the police powers, which, by the constitution, are reserved to the several States, and each State acts entirely independently of the others in its legislation upon these subjects. Sixteen of the States, namely, Alabama, California, Colorado, Florida, Illinois, Iowa, Minnesota, Missouri, Montana, New Jersey, New York, North Carolina, Oregon, South Carolina, Tennessee, and Virginia, have medical examining and licensing bodies, which do not give instruction.

In some of the States these bodies are the States Boards of Health, in others they are the State medical societies, in a few they are special Boards of physicians appointed for the purpose; in New York the Board of Regents of the University of the State of New York has charge of the matter, but its chief work is the enforcement of examinations of students as to preliminary education.

Most of the laws creating such independent examining and licensing bodies are of comparatively recent date, and, as they are usually not retroactive, have only sifted the recent applicants. The effect of the action of these Boards upon the medical schools has, however, already been very apparent. In the regular medical schools in the United States the percentage of graduates to matriculants was in 1881-2, 37·1; in 1888-9 it was 30·9. The number of matriculants in these schools in 1888-9 was 11·904, and the number of graduates was 3,681. The number of medical schools which now require attendance for three years upon a graded course of lectures has increased 25 per cent. within two years. The statistics given above relate exclusively to medical schools; in addition to these there are fourteen homœopathic, nine eclectic, and two physio-medical schools having about 1,800 students, and graduating about 650 M.D.'s yearly.

The proportion of physicians to population is much higher in the United States than it is in Europe, and it will be seen from the above figures that the number of aspirants is excessive.

The impulse to greater stringency in the requirements for licence to practice, and to improvements in medical education, has come mainly from the medical profession itself, and the opposition to such legislation has come chiefly from the homœpathic and other medical sects, which opposition has thus far, in many of the States, been effectual in preventing action.

While, as has been said, each State is independent of the others in its action on this subject, it is nevertheless true that those States which fix a higher standard of requirements do influence the curriculum of medical schools in other States. The average medical student is uncertain as to the locality in which he may settle, and prefers those schools whose diplomas are acceptable to State examining Boards to those which are not thus recognised. Moreover, each State which regulates the licensing to practise, in so far limits the field in which the poorly-educated M.D. can establish himself, and thus throws an increasing number of such men into the States which have not yet provided against them. It is, therefore, quite certain that in the course of time the natural laws of supply and demand will cause all the States not only to regulate the practice of medicine in some way, but to approach a comparatively uniform standard in this respect. What this standard will be is indicated by the regulation of the Illinois Board, that after 1891 graduates desiring to practise in that State must present evidence that they have studied medicine for four years, and attended at least three annual courses of lectures, which must include a course in hygiene and in medical jurisprudence, as well as in the ordinary branches.

No special certificates or degrees in surgery or in obstetrics are given in the medical schools—the degree is always that of M.D. In several of the large cities where there is abundance of clinical material, post-graduate schools of medicine have been established. Of these the most important is the New York Polytechnic, which was organized in 1880, and the New York Post-graduate Medical School, organized in 1882. None but practitioners are admitted, and there are no didactic lectures; the chief work is clinical medicine and surgery instruction, which goes on throughout the year.

There are six medical schools exclusively for women, and twenty-six which admit both sexes. Six are for coloured students.

The medical schools which have the highest standard as to preliminary education, and whose diplomas are at present probably the most valuable, are the Harvard Medical School in Boston, the College of Physicians and Surgeons in New York, and the University of Pennsylvania Medical Department in Philadelphia. These are the three oldest schools in the country, and all of them will soon require four years of attendance from their graduates.

The medical department of the Johns Hopkins University, in Baltimore, is not yet organized. There is a special course given in the University for those who propose to study medicine, and post-graduate instruction is given in the hospital, but the degree of M.D. is not granted. The complete organization of the medical department of this University will probably be effected within the next three years.

A very large number of American physicians have supplemented their two or three years' course of medical studies as undergraduates by post-graduate courses, by residence in hospitals for one or two years, and by one or more years' study in European schools. They travel more than our physicians do, and this is especially the case as regards the physicians in the large eastern cities. The men who are now attending the schools which require but two years' attendance are for the most part poor, and expect to practise in comparatively thinly settled districts, where the annual earnings at the best can be but small.

This view of the case is stated in a recent editorial in the "Memphis Medical Monthly" (a journal published at Memphis, Tennessee), as follows: "There are localities in almost every part of the country where neither the people nor the doctors enjoy a very high degree of either general or special education. It has been said that such classes of people prefer the grade of doctors they already have. It has come to our knowledge in no less than three instances that the poorly-educated doctor, with his breeches crammed in his boots and a glib tongue, has, with perfect ease, under such circumstances, routed the educated physician."

It would seem that the United States does not hold out flattering prospects to medical immigrants, and that an English physician should not expect to obtain there any more remunerative practice than that which he has at home. Those who are failures here will probably be still greater failures there.

